# Supplementary material for: Two-parameter Hong-Ou-Mandel dip
Source: Sci Rep. 2019 Jul 25;9:10821. doi: 10.1038/s41598-019-47207-3 (PMC6658540; doi:10.1038/s41598-019-47207-3)
Supplement: Supplementary file 1 — Supplementary Information for the revised manuscript [file 41598_2019_47207_MOESM1_ESM.pdf]

Supplementary Information

Two-parameter Hong-Ou-Mandel dip

Yu Yang[1,2], Luping Xu[1,*] and Vittorio Giovannetti[3]
[1]School of Aerospace Science and Technology, Xidian University, Xi'an 710126, China
[2]Scuola Normale Superiore, I-56126 Pisa, Italy
[3]NEST, Scuola Normale Superiore and Istituto Nanoscienze-CNR, I-56127 Pisa, Italy
*xidian_lpx@163.com

More on the HOM

Optimal CP states

One can easily verify that the CP state defined in equation (17) of main article is the only coherent pulse that ensures zero value of $R(\tau)$ for $\tau = 0$. Indeed consider a generalized multi-mode Coherent Pulses state $|\{\alpha_1, \alpha_2\}^{(a)}\rangle$ with asymmetric amplitudes $\alpha_1(\omega) \neq \alpha_2(\omega)$, in the two input arms of the interferometer, i.e.

$$\hat{a}_j(\omega)|\{\alpha_1,\alpha_2\}^{(a)}\rangle = \alpha_j(\omega)|\{\alpha_1,\alpha_2\}^{(a)}\rangle\ , \quad j = 1,2\ . \tag{1}$$

In terms of the output fields this yields

$$\begin{aligned} \hat{c}_1(\omega)|\{\alpha_1,\alpha_2\}^{(a)}\rangle &= \tfrac{\alpha_1(\omega)e^{-i\omega\tau}+\alpha_2(\omega)e^{i\omega\tau}}{\sqrt{2}}|\{\alpha_1,\alpha_2\}^{(a)}\rangle\ , \\ \hat{c}_2(\omega)|\{\alpha_1,\alpha_2\}^{(a)}\rangle &= \tfrac{\alpha_1(\omega)e^{-i\omega\tau}-\alpha_2(\omega)e^{i\omega\tau}}{\sqrt{2}}|\{\alpha_1,\alpha_2\}^{(a)}\rangle\ , \end{aligned} \tag{2}$$

and hence a coincidence counts

$$R(\tau) = \frac{1}{4}\left[(A_1+A_2)^2 - 4[\mathrm{Re}f(\tau)]^2\right]\ , \tag{3}$$

where for $j = 1,2$, $A_j := \int d\omega \left|\alpha_j(\omega)\right|^2$ is the total photon intensity at the j-th input port, and where

$$f(\tau) := \int d\omega \alpha_1(\omega)\alpha_2^*(\omega)e^{-2i\omega\tau}\ . \tag{4}$$

By Cauchy-Schwarz inequality it follows that, for all τ, the modulus of $f(\tau)$ is smaller or equal than $\sqrt{A_1A_2}$, i.e.

$$|f(\tau)|^2 \leq A_1A_2\ , \tag{5}$$

the bound being saturated for if and only if it exists a constant k such that

$$\alpha_1(\omega)e^{-i\omega\tau} = k\alpha_2(\omega)e^{i\omega\tau}\ , \tag{6}$$

holds true for all ω. Replacing this into Eq. (3) and using the fact that $[\mathrm{Re}f(\tau)]^2 \leq |f(\tau)|^2$ we finally get

$$R(\tau) \geq \frac{1}{4}(A_1 - A_2)^2\ . \tag{7}$$

Putting all this together it follows that to have $R(\tau) = 0$ for some τ, we need to fulfil Eq. (6) while ensuring that $A_1 = A_2$, i.e. having the k constant equal to 1 (up to an irrelevant global phase). In particular imposing $R(\tau) = 0$ for $\tau = 0$ this implies $\alpha_1(\omega) = \alpha_2(\omega) = \alpha(\omega)$, forcing $|\{\alpha_1,\alpha_2\}^{(a)}\rangle$ to coincide with the symmetric state $|\alpha^{(a)}\rangle$ (see the subsection "The dip with coherent pulses (CP) state" of main article).

Choice of the frequency spectrum function

In order to get a proper HOM dip the frequency amplitude $\Psi_S(\omega,\omega')$ of the bi-photon state has to be symmetric. To see this consider the case in which the input state of the HOM is given by the following BP state

$$|\Psi_A^{(a)}\rangle := \int d\omega \int d\omega' \Psi_A(\omega,\omega')\hat{a}_1^\dagger(\omega)\hat{a}_2^\dagger(\omega')|vac\rangle\ , \tag{8}$$

where now $\Psi_A(\omega,\omega') = -\Psi_A(\omega',\omega)$ is anti-symmetric under exchange of ω and ω'. Computing the final coincidence counts by the same way related with equations (8), (9) and (10) of main article, we have

$$R_{\text{BP}}(\tau) = \int d\nu \overline{P}_S(\nu)\cos^2(\nu\tau)\ , \tag{9}$$

where $\overline{P}_S(\nu) := \int d\nu' \left|\Psi_A(\nu'+\nu/2, \nu'-\nu/2)\right|^2$, which for $\tau = 0$ does not reach zero.

Losses in the HOM interferometer

Consider the case where the photons propagating along the path $\mathbf{A}_j$ of the standard HOM setting of Figure 1 a) of main article, undergoes to loss and dispersion effects characterized by a frequency dependent complex amplitude probability $\xi_j(\omega)$, with modulus $|\xi_j(\omega)|$ spanning from 1 (no loss) to 0 (complete absorption), which in principle may exhibit a non trivial dependence upon the path label j. Under these conditions equations (4) and (7) of main article rewrite as

$$\begin{aligned} \hat{c}_1(\omega) &= \tfrac{\xi_1(\omega)\hat{a}_1(\omega)e^{-i\omega\tau}+\xi_2(\omega)\hat{a}_2(\omega)e^{i\omega\tau}}{\sqrt{2}}+\dots\,, \\ \hat{c}_2(\omega) &= \tfrac{\xi_1(\omega)\hat{a}_1(\omega)e^{-i\omega\tau}-\xi_2(\omega)\hat{a}_2(\omega)e^{i\omega\tau}}{\sqrt{2}}+\dots\,, \end{aligned} \tag{10}$$

and

$$\begin{aligned} \hat{a}_1(\omega) &= e^{i\omega\tau}\xi_1^*(\omega)(\hat{c}_1(\omega)+\hat{c}_2(\omega))/\sqrt{2}+\dots\,, \\ \hat{a}_2(\omega) &= e^{-i\omega\tau}\xi_2^*(\omega)(\hat{c}_1(\omega)-\hat{c}_2(\omega))/\sqrt{2}+\dots\,, \end{aligned} \tag{11}$$

where the dots refer to the presence of vacuum terms which we do not need to define explicitly as they will not give a net contribution to the intensity-intensity term, i.e. equation (3) of main article. Accordingly, when expressing the input state in terms of the output field operators the component where one photon reaches detector D_1 and the other reaches detector D_2, is still as equation (9) of main article with the bi-photon amplitude $\Psi_A^{(\tau)}(\omega,\omega')$ being substituted by the function

$$\widetilde{\Psi}_A^{(\tau)}(\omega,\omega') = \Psi_S(\omega,\omega')[i\sin((\omega-\omega')\tau)\tfrac{\xi_1(\omega)\xi_2(\omega')+\xi_1(\omega')\xi_2(\omega)}{2} - \cos((\omega-\omega')\tau)\tfrac{\xi_1(\omega)\xi_2(\omega')-\xi_1(\omega')\xi_2(\omega)}{2}]\,. \tag{12}$$

The above replacement has a negative impact on the HOM dip: in particular unless $\xi_1(\omega)=\xi_2(\omega)$ the associated function $R_{\mathrm{BP}}(\tau)$ will no longer being able to reach zero for $\tau=0$. Assume however $\xi_j(\omega)$ to be sufficiently smooth in the integration domain where $\Psi_S(\omega,\omega')$ is significantly different from zero, in such a way that we can approximate them as

$$\xi_j(\omega) \simeq \xi_j(\omega_0)\,. \tag{13}$$

Under this assumption, Eq. (12) reduces to

$$\widetilde{\Psi}_A^{(\tau)}(\omega,\omega') \simeq i\xi_1(\omega_0)\xi_2(\omega_0)\Psi_S(\omega,\omega')\sin((\omega-\omega')\tau)\,, \tag{14}$$

which, even in the presence of strong asymmetries between the two paths (i.e. even when $\xi_1(\omega_0)\neq\xi_2(\omega_0)$), leads to a simple rescaling of the associated rate that does not affect neither the existence of the dip at $\tau=0$, nor its 100% visibility. For instance in the case of the Gaussian bi-photon spectrum (equation (14) of main article) we get

$$R_{\mathrm{BP}}(\tau) \simeq |\xi_1(\omega_0)\xi_2(\omega_0)|^2(1-e^{-2\,\Delta^2\Omega_-\tau^2})/2\,, \tag{15}$$

which a part from a global multiplication factor, has the same functional dependence upon τ of its lossless counterpart (equation (16) of main article).

The situation changes drastically however when injecting into the interferometer the semiclassical state $|\alpha^{(a)}\rangle$. Based on Eq. (10) in this case we get

$$\begin{aligned} \hat{c}_1(\omega)|\alpha^{(a)}\rangle &= \tfrac{\alpha(\omega)\left(\xi_1(\omega)e^{-i\omega\tau}+\xi_2(\omega)e^{i\omega\tau}\right)}{\sqrt{2}}|\alpha^{(a)}\rangle\,, \\ \hat{c}_2(\omega)|\alpha^{(a)}\rangle &= \tfrac{\alpha(\omega)\left(\xi_1(\omega)e^{-i\omega\tau}-\xi_2(\omega)e^{i\omega\tau}\right)}{\sqrt{2}}|\alpha^{(a)}\rangle\,, \end{aligned} \tag{16}$$

which inserted into equation (3) of main article and adopting the condition (13) yields

$$\begin{aligned} R_{\mathrm{CP}}(\tau) &\simeq \frac{A^2}{4}(|\xi_1(\omega_0)|^2+|\xi_2(\omega_0)|^2)^2\left[1-(1-\eta_{\mathbf{A}_1\mathbf{A}_2})\left(\int d\omega P_\alpha(\omega)\cos(2\omega\tau-\beta)\right)^2\right]\,, \\ &= \frac{A^2}{4}(|\xi_1(\omega_0)|^2+|\xi_2(\omega_0)|^2)^2\left[1-(1-\eta_{\mathbf{A}_1\mathbf{A}_2})\cos^2(2\omega_0\tau-\beta)e^{-4\,\Delta^2\omega\tau^2}\right]\,, \end{aligned} \tag{17}$$

the second identity being written under the Guassian spectrum assumption that led us to (equation (21) of main article). In the above expressions β is the phase of complex term $\xi_1(\omega_0)\xi_2(\omega_0)^*$ which can be used to account for asymmetries in the dispersion experienced by the signals propagating along the paths $\mathbf{A}_1$ and $\mathbf{A}_2$, while

$$\eta_{\mathbf{A}_1\mathbf{A}_2} := \left(\tfrac{|\xi_1(\omega_0)|^2-|\xi_2(\omega_0)|^2}{|\xi_1(\omega_0)|^2+|\xi_2(\omega_0)|^2}\right)^2\,, \tag{18}$$

gauges the relative difference between the corresponding losses. As evident from (19) having $\xi_1(\omega_0) \neq \xi_2(\omega_0)$ compromises both the possibility of having a zero for $\tau = 0$ due to the presence of the phase shift β, and the visibility of the signal by the presence of the factor $(1 - \eta_{\mathbf{A}_1\mathbf{A}_2})$. In particular, while under coarse graining (equation (22) of main article) the β dependence is washed away, the visibility reduction remains as evident from the expression

$$\overline{R}_{\text{CP}}(\tau) \simeq \frac{A^2}{4}(|\xi_1(\omega_0)|^2 + |\xi_2(\omega_0)|^2)^2 \left(1 - \tfrac{1-\eta_{\mathbf{A}_1\mathbf{A}_2}}{2} e^{-4\,\Delta^2\omega\tau^2}\right) , \tag{19}$$

which for $\eta_{\mathbf{A}_1\mathbf{A}_2} = 1$ (maximum path asymmetry) exhibits a completely flat, τ-independent, behaviour.

More on the modified HOM configuration

Decomposition of the output state

Similar with the decomposition for $|\Psi^{(c)}(\tau_1, \tau_2)\rangle$ in equation (28) of main article, $|\Phi^{(c)}(\tau_1, \tau_2)\rangle$ is also characterized by two components, i.e.

$$|\Phi^{(c)}(\tau_1, \tau_2)\rangle = |\Phi_+^{(c)}(\tau_1, \tau_2)\rangle + |\Phi_-^{(c)}(\tau_1, \tau_2)\rangle , \tag{20}$$

here

$$|\Phi_+^{(c)}(\tau_1, \tau_2)\rangle \quad := \quad \frac{1}{2}\int d\omega \int d\omega' \Phi_+^{(\tau_1,\tau_2)}(\omega, \omega')[c_1^\dagger(\omega)c_1^\dagger(\omega') + c_2^\dagger(\omega)c_2^\dagger(\omega')]|vac\rangle , \tag{21}$$

$$|\Phi_-^{(c)}(\tau_1, \tau_2)\rangle \quad := \quad \frac{1}{2}\int d\omega \int d\omega' \Phi_-^{(\tau_1,\tau_2)}(\omega, \omega')[c_1^\dagger(\omega)c_1^\dagger(\omega') - c_2^\dagger(\omega)c_2^\dagger(\omega')]|vac\rangle , \tag{22}$$

with

$$\Phi_+^{(\tau_1,\tau_2)}(\omega, \omega') \quad := \quad -ie^{i\theta}\Psi_S(\omega, \omega')\cos((\omega - \omega')\tau_1)\sin((\omega + \omega')\tau_2 + \theta) , \tag{23}$$

$$\Phi_-^{(\tau_1,\tau_2)}(\omega, \omega') \quad := \quad e^{i\theta}\Psi_S(\omega, \omega')\sin((\omega - \omega')\tau_1)\sin((\omega - \omega')\tau_2) . \tag{24}$$

The role of the achromatic phase shift

In order to understand the role played by the phase shift θ in our construction let us observe that a generic biphoton state propagating along two distinct optical paths (say $\mathbf{A}_1$ and $\mathbf{A}_2$) and characterized by annihilation operators $\hat{a}_1(\omega)$ and $\hat{a}_2(\omega)$ respectively, can always be expressed as the sum of four terms belonging to orthogonal classes, i.e.

$$|\Psi_S^{(a)}\rangle + |\Psi_A^{(a)}\rangle + |\Phi_+^{(a)}\rangle + |\Phi_-^{(a)}\rangle , \tag{25}$$

with

$$\langle\Psi_A^{(a)}|\Psi_S^{(a)}\rangle = \langle\Phi_+^{(a)}|\Phi_-^{(a)}\rangle = \langle\Psi_{S,A}^{(a)}|\Phi_\pm^{(a)}\rangle = 0 . \tag{26}$$

Here, according to the notation introduced in the subsection "The dip with frequency correlated bi-photon (BP) state" of main article,

$$|\Psi_{S,A}^{(a)}\rangle := \int d\omega \int d\omega' \Psi_{S,A}(\omega, \omega')\hat{a}_1^\dagger(\omega)\hat{a}_2^\dagger(\omega')|vac\rangle, \tag{27}$$

describe configurations with one photon in $\mathbf{A}_1$ and one photon in $\mathbf{A}_2$, with spectral amplitudes $\Psi_S(\omega, \omega')$ and $\Psi_A(\omega, \omega')$ that are, respectively, even and odd under exchange of the frequency ω and ω', while

$$|\Phi_\pm^{(a)}\rangle := \frac{1}{2}\int d\omega \int d\omega' \Phi_\pm(\omega, \omega')\big(\hat{a}_1^\dagger(\omega)\hat{a}_1^\dagger(\omega') \pm \hat{a}_2^\dagger(\omega)\hat{a}_2^\dagger(\omega')\big)|vac\rangle , \tag{28}$$

represent, respectively, states where the photons are in coherent $\pm$-superposition of states where they are either both in $\mathbf{A}_1$ or both in $\mathbf{A}_2$ (the associated amplitudes $\Phi_\pm(\omega, \omega')$ being always even under exchange of ω and ω').

Adopting the above decomposition we want now to study how a generic BP state is transformed under the action of the various element that compose our interferometer, i.e. the 50:50 beam-splitters, the delays elements, and the achromatic phase shift:

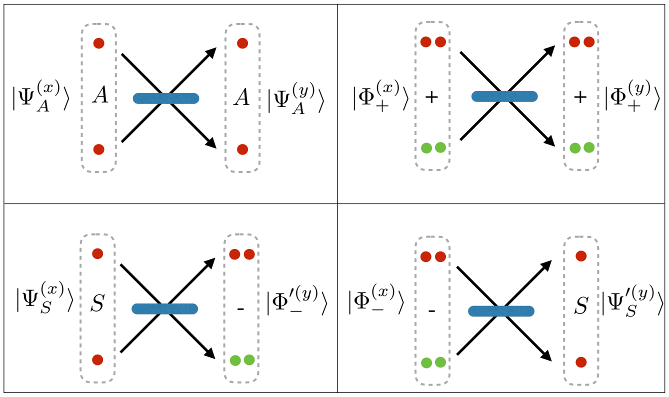

Figure 1. Graphical representation of the 50:50 beam splitter mapping (equations (30) and (32)).

- *Beam-splitter mapping:*– Suppose that the state (25) impinges into a 50:50 beam-splitter emerging into the paths $\mathbf{B}_1$ and $\mathbf{B}_2$ characterized by annihilation operators $\hat{b}_1(\omega)$ and $\hat{b}_2(\omega)$ and connected to $\hat{a}_1(\omega)$ and $\hat{a}_2(\omega)$ via the mapping

$$\begin{aligned} \hat{a}_1(\omega) &= (\hat{b}_1(\omega)+\hat{b}_2(\omega))/\sqrt{2}\,, \\ \hat{a}_2(\omega) &= (\hat{b}_1(\omega)-\hat{b}_2(\omega))/\sqrt{2}\,, \end{aligned} \tag{29}$$

see Fig. 1. By direct substitution it follows that $|\Psi_A^{(a)}\rangle$ and $|\Phi_+^{(a)}\rangle$ will produce output configurations that maintain the same structure,

$$|\Psi_A^{(a)}\rangle = |\Psi_A'^{(b)}\rangle\,, \qquad |\Phi_+^{(a)}\rangle = |\Phi_+'^{(b)}\rangle\,, \tag{30}$$

with amplitudes

$$\begin{aligned} \Psi_A'(\omega,\omega') &:= -\Psi_A(\omega,\omega')\,, \\ \Phi_+'(\omega,\omega') &:= \Phi_+(\omega,\omega')\,, \end{aligned} \tag{31}$$

while $|\Psi_S^{(a)}\rangle$ and $|\Phi_-^{(a)}\rangle$ will exchange their role, i.e.

$$|\Psi_S^{(a)}\rangle = |\Phi_-''^{(b)}\rangle\,, \qquad |\Phi_-^{(a)}\rangle = |\Psi_S''^{(b)}\rangle\,, \tag{32}$$

the associated amplitudes being

$$\begin{aligned} \Phi_-''(\omega,\omega') &:= \Psi_S(\omega,\omega')\,, \\ \Psi_S''(\omega,\omega') &:= \Phi_-(\omega,\omega')\,, \end{aligned} \tag{33}$$

respectively.

- *Delay elements:*– By the same token we notice that when instead the state (25) passes through a delay inducing the mapping

$$\begin{aligned} \hat{a}_1(\omega) &= e^{i\omega\tau}\hat{b}_1(\omega)\,, \\ \hat{a}_2(\omega) &= e^{-i\omega\tau}\hat{b}_2(\omega)\,, \end{aligned} \tag{34}$$

the components $|\Psi_{S,A}^{(a)}\rangle$ gets mixed according to the transformations

$$\begin{aligned} |\Psi_S^{(a)}\rangle &= |\Psi_S'^{(b)}\rangle + |\Psi_A'^{(b)}\rangle\,, \\ |\Psi_A^{(a)}\rangle &= |\Psi_S''^{(b)}\rangle + |\Psi_A''^{(b)}\rangle\,, \end{aligned} \tag{35}$$

with the new amplitudes given by

$$\begin{aligned}
\Psi'_S(\omega,\omega') &= \Psi_S(\omega,\omega')\cos((\omega-\omega')\tau) , \\
\Psi'_A(\omega,\omega') &= -i\Psi_S(\omega,\omega')\sin((\omega-\omega')\tau) , \\
\Psi''_S(\omega,\omega') &= -i\Psi_A(\omega,\omega')\sin((\omega-\omega')\tau) , \\
\Psi''_A(\omega,\omega') &= \Psi_A(\omega,\omega')\cos((\omega-\omega')\tau) .
\end{aligned} \tag{36}$$

Analogously the components $|\Phi^{(a)}_{\pm}\rangle$ evolve as in

$$\begin{aligned}
|\Phi^{(a)}_{+}\rangle &= |\Phi'^{(b)}_{+}\rangle + |\Phi'^{(b)}_{-}\rangle , \\
|\Phi^{(a)}_{-}\rangle &= |\Phi''^{(b)}_{+}\rangle + |\Phi''^{(b)}_{-}\rangle ,
\end{aligned} \tag{37}$$

with amplitudes

$$\begin{aligned}
\Phi'_{+}(\omega,\omega') &= \Phi_{+}(\omega,\omega')\cos((\omega+\omega')\tau) , \\
\Phi'_{-}(\omega,\omega') &= -i\Phi_{+}(\omega,\omega')\sin((\omega+\omega')\tau) , \\
\Phi''_{+}(\omega,\omega') &= -i\Phi_{-}(\omega,\omega')\sin((\omega+\omega')\tau) , \\
\Phi''_{-}(\omega,\omega') &= \Phi_{-}(\omega,\omega')\cos((\omega+\omega')\tau) .
\end{aligned} \tag{38}$$

- *Achromatic phase shift mapping:–* At variance the delay element analyzed in the previous paragraph, an achromatic phase shifter simply add a phase θ which is independent contribution to each of the frequency mode it acts on[1,2]. In other words indicating with $\hat{a}(\omega)$ and $\hat{b}(\omega)$ the corresponding input and output annihilation operators, the transformation we consider here connects them according to the mapping

$$\hat{a}(\omega) = e^{-i\theta}\hat{b}(\omega) . \tag{39}$$

Physically it can be realized by exploiting material which have a non-linear dispersion, or by merging slabs of birefringent waveplates. Assuming hence it operates selectively only the path $\mathbf{A}_2$, when acting on the BP state (25), it will preserve the structure of the $|\Psi^{(a)}_{S,A}\rangle$ terms, i.e.

$$|\Psi^{(a)}_{S,A}\rangle = |\Psi'^{(b)}_{S,A}\rangle , \tag{40}$$

simply adding a global phase to their amplitudes

$$\Psi'_{S,A}(\omega,\omega') := e^{i\theta}\Psi_{S,A}(\omega,\omega') . \tag{41}$$

On the contrary, when operating upon the $|\Phi^{(a)}_{\pm}\rangle$ terms it will induce a non trivial rotation of the form

$$\begin{aligned}
|\Phi^{(a)}_{+}\rangle &= |\Phi'^{(b)}_{+}\rangle + |\Phi'^{(b)}_{-}\rangle , \\
|\Phi^{(a)}_{-}\rangle &= |\Phi''^{(b)}_{+}\rangle + |\Phi''^{(b)}_{-}\rangle ,
\end{aligned} \tag{42}$$

where

$$\begin{aligned}
\Phi'_{+}(\omega,\omega') &= e^{i\theta}\Phi_{+}(\omega,\omega')\,\cos\theta , \\
\Phi'_{-}(\omega,\omega') &= -ie^{i\theta}\Phi_{+}(\omega,\omega')\,\sin\theta , \\
\Phi''_{+}(\omega,\omega') &= -ie^{i\theta}\Phi_{-}(\omega,\omega')\,\sin\theta , \\
\Phi''_{-}(\omega,\omega') &= e^{i\theta}\Phi_{-}(\omega,\omega')\,\cos\theta ,
\end{aligned} \tag{43}$$

which in particular for $\theta = \pi/2$ results in exchanging the spectral functions of $|\Phi^{(a)}_{+}\rangle$ and $|\Phi^{(a)}_{-}\rangle$.

It goes without mentioning that concatenating the above expressions one can easily show that indeed equation (8) of main article is the correct BP state emerging from the conventional HOM interferometer when injecting the input state equation (5) of main article. Iterating twice the same procedure we can as well describe the evolution under the modified HOM setting. Specifically, let us consider first the case where $\theta = 0$, i.e. when the achromatic shift is not present in the scheme. Starting from the state $|\Psi_S^{(a)}\rangle$ of equation (5) of main article, after the first time delay τ_1 and the first beam splitter we get

$$|\Psi_A^{(b)}(\tau_1)\rangle + |\Phi_-^{(b)}(\tau_1)\rangle \;, \tag{44}$$

having probability amplitudes $i\Psi_S(\omega,\omega')\sin((\omega-\omega')\tau_1)$ and $\Psi_S(\omega,\omega')\cos((\omega-\omega')\tau_1)$ respectively. Thanks to Eq. (38), just before it reaches the second beam splitter, the delay τ_2 maps $|\Psi_A^{(b)}(\tau_1)\rangle$ into the superposition

$$|\Psi_A^{(b)}(\tau_1,\tau_2)\rangle + |\Psi_S^{(b)}(\tau_1,\tau_2)\rangle \;, \tag{45}$$

characterized by the amplitudes which now write as $i\Psi_S(\omega,\omega')\sin((\omega-\omega')\tau_1)\cos((\omega-\omega')\tau_2)$ and $\Psi_S(\omega,\omega')\sin((\omega-\omega')\tau_1)\sin((\omega-\omega')\tau_2)$; also it sends $|\Phi_-^{(b)}(\tau_1)\rangle$ into

$$|\Phi_+^{(b)}(\tau_1,\tau_2)\rangle + |\Phi_-^{(b)}(\tau_1,\tau_2)\rangle \;, \tag{46}$$

the first having amplitude $-i\Psi_S(\omega,\omega')\cos((\omega-\omega')\tau_1)\sin((\omega+\omega')\tau_2)$ and the second having instead $\Psi_S(\omega,\omega')\cos((\omega-\omega')\tau_1)\cos((\omega+\omega')\tau_2)$. Accordingly the state impinging at the input of the second beam splitter writes as the sum of four terms that undergoes the following transformation

$$\begin{array}{c} |\Psi_A^{(b)}(\tau_1,\tau_2)\rangle + |\Psi_S^{(b)}(\tau_1,\tau_2)\rangle + |\Phi_+^{(b)}(\tau_1,\tau_2)\rangle + |\Phi_-^{(b)}(\tau_1,\tau_2)\rangle \\ \downarrow \text{(second beam splitter)} \downarrow \\ |\Psi_A^{(c)}(\tau_1,\tau_2)\rangle + |\Phi_-^{(c)}(\tau_1,\tau_2)\rangle + |\Phi_+^{(c)}(\tau_1,\tau_2)\rangle + |\Psi_S^{(c)}(\tau_1,\tau_2)\rangle \;, \end{array} \tag{47}$$

with final amplitudes given by the expressions

$$\begin{aligned} \Psi_A^{(\tau_1,\tau_2)}(\omega,\omega') &= -i\Psi_S(\omega,\omega')\sin((\omega-\omega')\tau_1)\cos((\omega-\omega')\tau_2) \;, \\ \Phi_-^{(\tau_1,\tau_2)}(\omega,\omega') &= \Psi_S(\omega,\omega')\sin((\omega-\omega')\tau_1)\sin((\omega-\omega')\tau_2) \;, \\ \Phi_+^{(\tau_1,\tau_2)}(\omega,\omega') &= -i\Psi_S(\omega,\omega')\cos((\omega-\omega')\tau_1)\sin((\omega+\omega')\tau_2) \;, \\ \Psi_S^{(\tau_1,\tau_2)}(\omega,\omega') &= \Psi_S(\omega,\omega')\cos((\omega-\omega')\tau_1)\cos((\omega+\omega')\tau_2) \;, \end{aligned} \tag{48}$$

(notice that the above equations coincide with the expression (28) of main article given in the main text for $\theta = 0$). Now for $\tau_1 = \tau_2 = 0$ we observe that all the terms nullify but for the last which instead reduces to the same state we had as input of the setup, i.e.

$$|\Psi_S^{(c)}(\tau_1 = 0, \tau_2 = 0)\rangle\Big|_{\theta=0} = \int d\omega \int d\omega' \Psi_S(\omega,\omega')\hat{c}_1^\dagger(\omega)\hat{c}_2^\dagger(\omega')|vac\rangle \;. \tag{49}$$

This will give a non zero contribution to the coincide rate, preventing one to get an absolute minimum at origin of the (τ_1,τ_2) plane.

The presence of the achromatic shift is introduced exactly to avoid this unfortunate event. The way it does it is by simply exchanging the role of $|\Phi_+^{(b)}(\tau_1,\tau_2)\rangle$ and $|\Phi_-^{(b)}(\tau_1,\tau_2)\rangle$ just before the action of the last beam splitter, which ultimately corresponds to exchanging the spectral functions of the output terms $|\Phi_+^{(c)}(\tau_1,\tau_2)\rangle$ and $|\Psi_S^{(c)}(\tau_1,\tau_2)\rangle$. Indeed as it commutes with the τ_2-delay element we can apply the achromatic shift at the level of the first line of Eq. (47). According to Eqs. (40) and (42) for $\theta = \pi/2$, apart from adding an irrelevant phase to both $|\Psi_S^{(b)}(\tau_1,\tau_2)\rangle$ and $|\Psi_A^{(b)}(\tau_1,\tau_2)\rangle$, this will induce a swap between the spectral functions of the terms $|\Phi_+^{(b)}(\tau_1,\tau_2)\rangle$ and $|\Phi_-^{(b)}(\tau_1,\tau_2)\rangle$, which now becomes $\Psi_S(\omega,\omega')\cos((\omega-\omega')\tau_1)\cos((\omega+\omega')\tau_2)$ for $|\Phi_+^{(b)}(\tau_1,\tau_2)\rangle$ and $-i\Psi_S(\omega,\omega')\cos((\omega-\omega')\tau_1)\sin((\omega+\omega')\tau_2)$ for $|\Phi_-^{(b)}(\tau_1,\tau_2)\rangle$. Accordingly the final state after the second beam splitter still has the form

$$|\Psi_A^{(c)}(\tau_1,\tau_2)\rangle + |\Phi_-^{(c)}(\tau_1,\tau_2)\rangle + |\Phi_+^{(c)}(\tau_1,\tau_2)\rangle + |\Psi_S^{(c)}(\tau_1,\tau_2)\rangle \;, \tag{50}$$

but has amplitudes that now are given by the expressions

$$\begin{aligned}
\Psi_A^{(\tau_1,\tau_2)}(\omega,\omega') &= \Psi_S(\omega,\omega')\sin((\omega-\omega')\tau_1)\cos((\omega-\omega')\tau_2)\,,\\
\Phi_-^{(\tau_1,\tau_2)}(\omega,\omega') &= i\Psi_S(\omega,\omega')\sin((\omega-\omega')\tau_1)\sin((\omega-\omega')\tau_2)\,,\\
\Phi_+^{(\tau_1,\tau_2)}(\omega,\omega') &= \Psi_S(\omega,\omega')\cos((\omega-\omega')\tau_1)\cos((\omega+\omega')\tau_2)\,,\\
\Psi_S^{(\tau_1,\tau_2)}(\omega,\omega') &= -i\Psi_S(\omega,\omega')\cos((\omega-\omega')\tau_1)\sin((\omega+\omega')\tau_2)\,,
\end{aligned} \tag{51}$$

which shows that, a part from irrelevant multiplicative constants, the spectrum of $|\Phi_+^{(c)}(\tau_1,\tau_2)\rangle$ and $|\Psi_S^{(c)}(\tau_1,\tau_2)\rangle$ have been exchanged with respect to the $\theta=0$ case. Accordingly for $\tau_1=\tau_2=0$ the final state gets

$$\begin{aligned}
|\Psi_S^{(c)}(\tau_1=0,\tau_2=0)\rangle\Big|_{\theta=\pi/2} &= |\Phi_+^{(c)}(\tau_1=0,\tau_2=0)\rangle\\
&= \frac{1}{2}\int d\omega\int d\omega'\Psi_S(\omega,\omega')\big(\hat{c}_1^\dagger(\omega)\hat{c}_1^\dagger(\omega')+\hat{c}_2^\dagger(\omega)\hat{c}_2^\dagger(\omega')\big)|vac\rangle\,,
\end{aligned} \tag{52}$$

which gives zero coincidences as anticipated.

Losses for the modified HOM interferometer

Adopting the same convention of the subsection "Losses in the HOM interferometer" above, in the presence of losses we can rewrite equations (24) and (26) of main article as

$$\begin{aligned}
\hat{b}_1(\omega) &= \tfrac{\xi_1(\omega)\hat{a}_1(\omega)e^{-i\omega\tau_1}+\xi_2(\omega)\hat{a}_2(\omega)e^{i\omega\tau_1}}{\sqrt{2}}+\dots\,,\\
\hat{b}_2(\omega) &= \tfrac{\xi_1(\omega)\hat{a}_1(\omega)e^{-i\omega\tau_1}-\xi_2(\omega)\hat{a}_2(\omega)e^{i\omega\tau_1}}{\sqrt{2}}+\dots\,,
\end{aligned} \tag{53}$$

and

$$\begin{aligned}
\hat{c}_1(\omega) &= \tfrac{\chi_1(\omega)\hat{b}_1(\omega)e^{-i\omega\tau_2}+\chi_2(\omega)\hat{b}_2(\omega)e^{i(\omega\tau_2+\theta)}}{\sqrt{2}}+\dots\,,\\
\hat{c}_2(\omega) &= \tfrac{\chi_1(\omega)\hat{b}_1(\omega)e^{-i\omega\tau_2}-\chi_2(\omega)\hat{b}_2(\omega)e^{i(\omega\tau_2+\theta)}}{\sqrt{2}}+\dots\,,
\end{aligned} \tag{54}$$

where, as anticipated into the main text $\xi_j(\omega)$ and $\chi_j(\omega)$ represent the absorption amplitudes associated with the photon propagating along the path $\mathbf{A}_j$ and $\mathbf{B}_j$. Accordingly equation (27) of main article can hence be re-expressed by

$$\begin{aligned}
\hat{c}_1(\omega) &= c(\omega\tau_2)\xi_1(\omega)\hat{a}_1(\omega)e^{-i\omega\tau_1}+s(\omega\tau_2)\xi_2(\omega)\hat{a}_2(\omega)e^{i\omega\tau_1}+\dots\,,\\
\hat{c}_2(\omega) &= s(\omega\tau_2)\xi_1(\omega)\hat{a}_1(\omega)e^{-i\omega\tau_1}+c(\omega\tau_2)\xi_2(\omega)\hat{a}_2(\omega)e^{i\omega\tau_1}+\dots\,,
\end{aligned} \tag{55}$$

with

$$\begin{aligned}
c(\omega\tau_2) &:= \frac{\chi_1(\omega)e^{-i\omega\tau_2}+\chi_2(\omega)e^{i(\omega\tau_2+\theta)}}{2}\,,\\
s(\omega\tau_2) &:= \frac{\chi_1(\omega)e^{-i\omega\tau_2}-\chi_2(\omega)e^{i(\omega\tau_2+\theta)}}{2}\,.
\end{aligned} \tag{56}$$

Using this expression to write the BP input state in terms of the output mode operators, we then observe that its component associated with one photon per each output ports has the form

$$|\Psi^{(c)}(\tau_1,\tau_2)\rangle = |\Psi_A^{(c)}(\tau_1,\tau_2)\rangle+|\Psi_S^{(c)}(\tau_1,\tau_2)\rangle\,, \tag{57}$$

where

$$|\Psi_{A,S}^{(c)}(\tau_1,\tau_2)\rangle := \int d\omega\int d\omega'\Psi_{A,S}^{(\tau_1,\tau_2)}(\omega,\omega')\hat{c}_1^\dagger(\omega)\hat{c}_2^\dagger(\omega')|vac\rangle$$

with spectral amplitudes

$$\begin{aligned}
\Psi_A^{(\tau_1,\tau_2)}(\omega,\omega') &\cong \Psi_S(\omega,\omega')(-i)e^{i\theta}\xi_1(\omega_0)\xi_2(\omega_0)\chi_1(\omega_0)\chi_2(\omega_0)\sin((\omega-\omega')\tau_1)\cos((\omega-\omega')\tau_2)\,,\\
\Psi_S^{(\tau_1,\tau_2)}(\omega,\omega') &\cong \frac{1}{2}\Psi_S(\omega,\omega')e^{i\theta}\xi_1(\omega_0)\xi_2(\omega_0)\cos((\omega-\omega')\tau_1)\left\{[\chi_1^2(\omega_0)+\chi_2^2(\omega_0)]\cos((\omega+\omega')\tau_2+\theta)\right.\\
&\quad \left.-i[\chi_1^2(\omega_0)-\chi_2^2(\omega_0)]\sin((\omega+\omega')\tau_2+\theta)\right\}\,,
\end{aligned} \tag{58}$$

that we report under the white-noise assumption equation (43) of main article. Accordingly from equation (32) of main article we finally get the following expression for the rate

$$R_{\text{BP}}(\tau_1,\tau_2) = \langle\Psi_A^{(c)}(\tau_1,\tau_2)|\Psi_A^{(c)}(\tau_1,\tau_2)\rangle + \langle\Psi_S^{(c)}(\tau_1,\tau_2)|\Psi_S^{(c)}(\tau_1,\tau_2)\rangle\ , \tag{59}$$

where

$$\begin{aligned}
\langle\Psi_A^{(c)}(\tau_1,\tau_2)|\Psi_A^{(c)}(\tau_1,\tau_2)\rangle &= \tfrac{|\xi_1(\omega_0)\xi_2(\omega_0)\chi_1(\omega_0)\chi_2(\omega_0)|^2}{8} \\
&\quad\times\left\{2+2e^{-2\Delta^2\Omega_-\tau_2^2}-2e^{-2\Delta^2\Omega_-\tau_1^2}-e^{-2\Delta^2\Omega_-(\tau_1+\tau_2)^2}-e^{-2\Delta^2\Omega_-(\tau_1-\tau_2)^2}\right\} \\
\langle\Psi_S^{(c)}(\tau_1,\tau_2)|\Psi_S^{(c)}(\tau_1,\tau_2)\rangle &= \tfrac{|\xi_1(\omega_0)\xi_2(\omega_0)|^2}{8}(1+e^{-2\Delta^2\Omega_-\tau_1^2}) \\
&\quad\times\left\{e^{-8\Delta^2\Omega_+\tau_2^2}\cos(4\omega_0\tau_2+2\theta)[(C+C^*)-i(C-C^*)]+|\chi_1(\omega_0)|^4+|\chi_2(\omega_0)|^4\right\},
\end{aligned} \tag{60}$$

with $C := (\chi_1(\omega_0)\chi_2^*(\omega_0))^2$, which under coarse graining yields equation (44) of main article.

The BP performance should be compared with the one obtained for the CP state. According to equation (3) of main article to get the following $R_{CP}(\tau_1,\tau_2)$ under the same white-noise assumption equation (43) of main article, this can be written as

$$R_{\text{CP}}(\tau_1,\tau_2) = \frac{A^2}{16}\Big(X^2(\tau_1,\tau_2)-Y^2(\tau_1,\tau_2)\Big)\ , \tag{61}$$

with

$$\begin{aligned}
X(\tau_1,\tau_2) &:= (|\chi_1|^2+|\chi_2|^2)(|\xi_1|^2+|\xi_2|^2)+e^{-2\Delta^2\omega\tau_1^2}(|\chi_1|^2-|\chi_2|^2)[\xi_1\xi_2^*e^{-2i\omega_0\tau_1}+h.c.]\ , \\
Y(\tau_1,\tau_2) &:= e^{-2\Delta^2\omega\tau_2^2}(|\xi_1|^2-|\xi_2|^2)[\chi_1\chi_2^*e^{-i(2\omega_0\tau_2+\theta)}+h.c.]-e^{-2\Delta^2\omega(\tau_1+\tau_2)^2}[\xi_1\xi_2^*\chi_1\chi_2^*e^{-2i\omega_0(\tau_1+\tau_2)}e^{-i\theta}+h.c.] \\
&\quad +e^{-2\Delta^2\omega(\tau_1-\tau_2)^2}[\xi_1\xi_2^*\chi_2\chi_1^*e^{-2i\omega_0(\tau_1-\tau_2)}e^{i\theta}+h.c.]\ ,
\end{aligned} \tag{62}$$

where for easy of notation we dropped the explicit ω_0 dependence in the writing of the χ_j and ξ_j parameters.

References

1. Pancharatnam, S. Achromatic combinations of birefringent plates-Part II. An achromatic quarter-wave plate. *Proc. Indian Acad. Sci.* **41,** 137-144 (1955).
2. Vilas, J. L., Sanchez-Brea, L. M. & Bernabeu, E. Optimal achromatic wave retarders using two birefringent wave plates. *Appl. Optics* **52,** 1892-1896 (2013).
